# GABA Metabolism, Transport and Their Roles and Mechanisms in the Regulation of Abiotic Stress (Hypoxia, Salt, Drought) Resistance in Plants

**DOI:** 10.3390/metabo13030347

**Published:** 2023-02-26

**Authors:** Ding Yuan, Xiaolei Wu, Binbin Gong, Ruixiao Huo, Liran Zhao, Jingrui Li, Guiyun Lü, Hongbo Gao

**Affiliations:** Collaborative Innovation Center of Vegetable Industry in Hebei, Key Laboratory of North China Water-Saving Irrigation Engineering, College of Horticulture, Hebei Agricultural University, Baoding 071000, China

**Keywords:** γ- aminobutyric acid, distribution, biosynthesis and catabolism, transporter from intracellular and extracellular, plant growth and development

## Abstract

γ- Aminobutyric acid (GABA) is a ubiquitous four-carbon non-protein amino acid. In plants, GABA is found in different cell compartments and performs different metabolic functions. As a signalling molecule, GABA participates in the regulation of tolerance to various abiotic stresses. Many research studies have found that GABA accumulates in large amounts when plants are subjected to abiotic stress, which have been demonstrated through the Web of Science, PubMed, Elsevier and other databases. GABA enhances the tolerance of plants to abiotic stress by regulating intracellular pH, ion transport, activating antioxidant systems and scavenging active oxygen species. In the process of GABA playing its role, transport is very important for the accumulation and metabolism pathway of GABA in cells. Therefore, the research on the transport of GABA across the cell membrane and the organelle membrane by transport proteins is a direction worthy of attention. This paper describes the distribution, biosynthesis and catabolism of GABA in plants. In addition, we focus on the latest progress in research on the transport of exogenous GABA and on the function and mechanism in the regulation of the abiotic stress response. Based on this summary of the role of GABA in the resistance to various abiotic stresses, we conclude that GABA has become an effective compound for improving plant abiotic tolerance.

## 1. Introduction

Climate change and human activities cause adverse conditions that severely hinder plant growth and reduce crop yields and food security, thus affecting the sustainable development of agriculture [1,2]. γ- Aminobutyric acid (GABA) is a ubiquitous four-carbon non-reducing amino acid found in prokaryotes and eukaryotes, including bacteria, fungi, plants and animals. GABA was first discovered in potato tuber tissue in 1949 [3], and subsequent studies have revealed its biosynthesis and related functions in plants and animals. GABA is mainly synthesized in the cytoplasm and is catabolized in mitochondria [4,5,6]. GABA synthesis and catabolism are regulated by many factors and play an important role in maintaining carbon and nitrogen balance [7,8], promoting plant photosynthesis [9,10], quenching reactive oxygen species (ROS) and other processes [11,12]. As a signalling molecule, GABA participates in the regulation of tolerance to various abiotic stresses, such as hypoxia [13], salinity [14] and drought [15]. For example, GABA can participate in salt stress resistance by promoting the antioxidant defence system and drought stress resistance through the regulation of stomatal aperture [16,17], ultimately promoting plant growth and increasing shelf-life [18] and crop storage quality [19,20]. In recent years, the transmembrane transport of GABA in plants has attracted much attention, and the process of transport from the apoplast to the cytoplasm and from the cytoplasm to various organelles is a popular research topic [21]. In this paper, we review the distribution, synthesis, catabolism and related transport of GABA in cells and discuss the progress in research on the role of GABA in the abiotic stress response.

## 2. Distribution of GABA in Plant Cells

**Figure 1:**
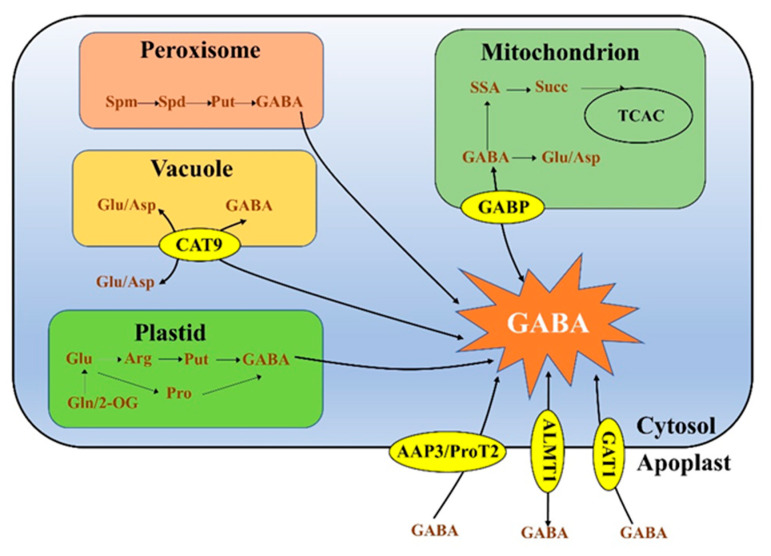
Model of distribution and transportation of GABA in plant cells.

GABA is widely distributed in the cytoplasm and various organelles of plant cells, such as mitochondria, vacuoles, peroxisomes and other plastids [1,17] (Figure 1). However, GABA performs different metabolic processes in different organelles. GABA catalysed by glutamate decarboxylase is found in the cytoplasm [22,23]. In mitochondria, GABA is converted to succinate through the GABA shunt [24] and ultimately enters the tricarboxylic acid (TCA) cycle or another metabolic pathway to produce γ- hydroxybutyric acid [25,26]. In addition, GABA can also be converted to glutamate and aspartate (Asp) in the mitochondria [27]. GABA produced by the polyamine degradation pathway is distributed in peroxisomes, where the transformation from spermidine to spermine and from spermine to putrescine takes place [28,29]. Johnson et al. [30] found that the amount of amino acids (including GABA) accumulated in tomato fruit was relatively high and mainly stored in the vacuole. GABA is also distributed in some plastids, in which glutamine (Gln) and 2-ketoglutarate (2-OG) are synthesized by glutamine synthetase and ferredoxin-dependent glutamate synthetase, respectively [31], after which GABA is generated by two reactions: in one reaction, glutamate generates arginine through the urea reaction, arginine decarboxylates to form putrescine, and putrescine generates GABA through copper amine oxidase and aldehyde dehydrogenase (ALDH10A8) [32]; in the other reaction, glutamate is converted to proline through ∆ 1-pyrroline-5-carboxylate synthetase and ∆ 1-pyrroline-5-carboxylate reductase, and then proline decarboxylates spontaneously to form pyrrolidine-1-yl (Pyr·), which is easily converted to Δ 1-pyrroline/4-aminobutyraldehyde; these products are then converted to GABA under the action of aldehyde dehydrogenase [33].

## 3. GABA Biosynthesis and Catabolism in Plants

**Figure 2:**
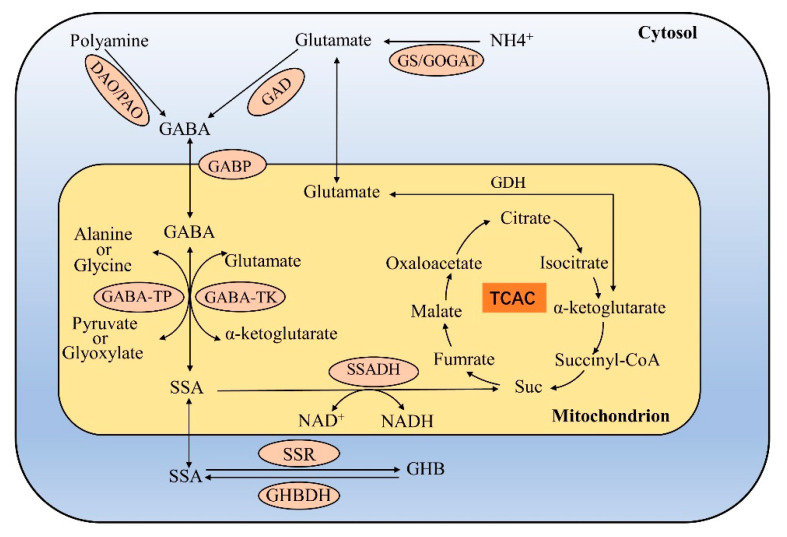
Model of biosynthesis and catabolism of GABA in plants.

Endogenous GABA in plants is mainly formed in two ways. The first is by the irreversible reaction of glutamate decarboxylation catalysed by glutamate decarboxylase (GAD) in the cytoplasm [22]. In addition, proline is also a potential indirect source of GABA in the cytoplasm [34]; however, there is currently no direct experimental evidence that GABA in plants is generated through proline. The other pathway involves polyamine degradation [35]. GABA mainly enters mitochondria for catabolism. There are also two GABA catabolism pathways. One is the common GABA shunt, which eventually results in the generation of succinate and enters the TCA cycle. The other is the participation of succinate reductase (SSR) in the catabolism of succinic semialdehyde (SSA) [36], and the end product is γ- hydroxybutyric acid (GHB) (Figure 2). 

### 3.1. Biosynthesis of GABA from Glutamate Decarboxylation, Polyamine Degradation and Proline Nonenzymatic Conversion

#### 3.1.1. Glutamate Decarboxylation

In the cytoplasm, glutamate undergoes an irreversible decarboxylation reaction under the action of glutamate decarboxylase to synthesize GABA [23], which is affected by glutamate concentration, adverse environmental conditions, pH and the expression of related genes.

Glutamate is the precursor of GABA and is converted to GABA through a decarboxylation reaction, so there is a direct relationship between glutamate content and GABA content. The production of glutamate in plants occurs in different cell regions. In the cytoplasm, nitrogen in ammonium ions can be assimilated into glutamate and other amino acids through the glutamine synthetase/glutamate synthetase (GS/GOGAT) cycle. Gln and 2-OG can also produce glutamate in plastids [37].

Another key factor affecting GABA synthesis is adverse environmental conditions. Adverse environmental conditions can stimulate an increase in intracellular calcium (Ca^2+^) levels [38,39]. Intracellular Ca^2+^ can induce the expression of the calmodulin (CaM) gene [40], generate CaM protein and form a Ca^2+^/CaM active complex [41,42,43]. The complex can activate the activity of GAD in vitro by binding with the C-terminal domain (the optimum pH is 7.0–7.5) [44,45], thus accelerating the stimulation of GABA biosynthesis. In addition, stress can promote the generation of hydrogen ions (H^+^) in the cytoplasm, which also helps to activate the activity of GAD [6], thus stimulating the biosynthesis of GABA [46].

*GAD* is the key GABA synthesis gene [47], and it has been identified in many species to date [48,49] (Table 1). *GAD1* is found in Arabidopsis, tomato and citrus. In Arabidopsis, *AtGAD1* is related to the synthesis of GABA, which mainly affects the level of GABA in roots and promotes an increase in glutamate levels [50]. In tomato, *SlGAD1* is related to fruit growth and development, but it is not significantly related to GABA content in fruit. In citrus, *CiGAD1* is closely related to GABA accumulation [51]. In rice, *OsGAD2* is crucial to the accumulation of GABA, showing high activity both in vivo and in vitro. The activity of *OsGAD2* in transgenic plants is more than 40 times higher than that in wild-type (WT) plants [52]. During the growth, development and ripening of tomato fruit, *SlGAD2* significantly increased the content of GABA in the fruit. *GAD3* has also been identified in tomato. Similar to *SlGAD2*, *SlGAD3* also increased the level of GABA in tomato fruit. *GAD4* has also been identified in Arabidopsis and tomato; however, it was shown that *AtGAD4* has no effect on the level of GABA, and *SlGAD4* is not related to the growth or development of tomato [45,48]. 

#### 3.1.2. Polyamine Degradation

Another GABA synthesis pathway is polyamine degradation. Polyamines (PAs) include putrescine (Put), spermine (Spm) and spermidine (Spd), of which Put is the central substance of polyamine biological metabolism and is a primary polyamine, and Spd and Spm are secondary and tertiary polyamines, respectively [53]. Put can be produced by ornithine, catalysed by ornithine decarboxylase (ODC) or arginine catalysed by arginine decarboxylase (ADC). Diamine oxidase (DAO) and polyamine oxidase (PAO) are amine oxidases [54]. The polyamine degradation pathway refers to the pathway through which diamines or polyamines (PAs) are catalysed by DAO and FAD-dependent PAO [55], respectively, to produce 4-aminobutyraldehyde (ABAL) and then dehydrogenated by 4-aminobutyraldehyde dehydrogenase (AMADH) to produce GABA [31]. 

Enzyme activity is a key factor affecting the degradation of polyamines to produce GABA. Three enzymes (DAO, PAO and AMADH) play a role in the degradation of polyamines [56]. Both subunits of DAO contain copper ions (Cu^2+^), so Cu^2+^ treatment can significantly improve the activity of DAO, while ethylenediaminetetraacetie acid (EDTA) treatment can reduce DAO activity [57]. PAO is highly dependent on flavin adenine dinucleotide (FAD), so quinine can strongly inhibit its activity. AMADH is an aldehyde dehydrogenase that uses nicotinamide adenine dinucleotide (NAD+) as a coenzyme [58,59]. 

Adverse environmental conditions can also affect the efficiency of polyamine degradation to generate GABA, which is mainly caused by increasing polyamine content. For example, in broad bean, anaerobic stress can induce an increase in the key enzyme activity of polyamine synthesis and promote the accumulation of polyamines [60,61], and in soybean roots, salt stress can increase the accumulation of free polyamines [62]. Adverse environmental conditions can also increase the activity of polyamine oxidase and promote the synthesis and accumulation of GABA through the polyamine degradation pathway [63,64,65,66].

Notably, although the polyamine degradation pathway is considered another important pathway for GABA synthesis, its ability to synthesize GABA in monocotyledons is far lower than that of the GABA shunt [58].

#### 3.1.3. Polyamine Degradation

In plastids, proline can react with hydroxyl radicals, which attack the N atom of proline, resulting in the simultaneous elimination of its own and the hydrogen ion (H^+^) located on the amino group of glutamate [34]. Hydrogen abstraction leads to the decarboxylation of proline to form pyrrolidin-1-yl. Then, pyrrolidin-1-yl continues to react with hydroxyl radicals to form Δ 1-pyrroline, which produces GABA under the action of pyrroline dehydrogenase (PYRR-DH) [67].

Stress is the key factor that affects the nonenzymatic conversion of proline to GABA because plants accumulate a large number of ROS under stress conditions, and hydroxyl radical, the most active ROS, is the main substance involved in the conversion of proline to GABA [68,69].

### 3.2. GABA Catabolism Generates Succinate and γ- Hydroxybutyric Acid (GHB)

#### 3.2.1. GABA Is Converted to Succinate

The conversion of GABA to succinate occurs in the mitochondrial matrix [70]. GABA is converted to succinic semialdehyde (SSA) by GABA transaminase (GABA-T), and then SSA can be oxidized by NAD^+^ dependent succinic semialdehyde dehydrogenase (SSADH) and eventually converted to succinate for entry into the TCA cycle [71]. This process is known as the GABA shunt [72]. Through the GABA shunt, GABA bypasses the TCA cycle in two steps from α-ketoglutarate through succinate CoA to succinate. In the conversion of GABA to SSA, two GABA-Ts (GABA-TK and GABA-TP) are mainly involved in the reaction [73]. GABA-TK uses α-ketoglutarate as an amino receptor to produce glutamate, and GABA-TP uses pyruvate as an amino receptor to produce alanine. GABA-TP also has GABA-TG activity; that is, glyoxylic acid is an amino receptor that produces glycine [74]. All of these reactions are reversible. 

Adverse environmental conditions can affect GABA catabolism. The ratio of NAD+ to NADH is low under stress, which inhibits the activity of SSADH and leads to the accumulation of SSA [75]. The accumulation of SSA in turn reduces the activity of GABA-T and eventually inhibits the catabolism of GABA. In addition, GABA-TK mainly exists in animals, yeast and fungi [76]. GABA-TP plays a major role in plants, and the gene associated with GABA-TP has been identified in tobacco and Arabidopsis. The optimal pH of GABA-TP is nine [75,77]; therefore, pH may be another factor affecting GABA catabolism in plants.

Many key genes are involved in the catabolism of GABA, and they are found in different locations of cells and perform related metabolic reactions to affect the catabolism pathway of GABA (Table 1). The *GABA-T* gene encodes GABA transaminase, which converts GABA into succinate semialdehyde [78]. In Arabidopsis, a *GABA-T* gene has been identified— *POP2* (*pollen-pistil incompatibility2*). *AtPOP2* produces a functional GABA-T enzyme that ensures the GABA gradient required to guide the growth of pollen tubes in the pistil, and the development of roots and shoots is regulated [63,79]. Three *GABA-T* (*SlGABA-T1*, *SlGABA-T2* and *SlGABA-T3*) genes have been identified in tomato. The expression of *SlGABA-T1* is higher than that of *SlGABA-T2* and *SlGABA-T3* during tomato fruit ripening. *SlGABA-T1* plays an important role in the metabolic pathway of the GABA shunt. This gene is mainly found in the mitochondrial matrix and encodes the GABA-T enzyme to ensure the conversion of GABA, while avoiding plant dwarfing and sterility. *SlGABA-T3* is mainly expressed in plastids to ensure the normal growth and development of plants [80]. *SlSSADH* and *AtSSADH* have been identified in tomato and Arabidopsis, respectively. By encoding succinic semialdehyde dehydrogenase to convert succinic semialdehyde into succinate, these genes affect GABA catabolism [17].

#### 3.2.2. GABA Is Converted to GHB

γ- Hydroxybutyric acid (GHB) is another product of GABA catabolism. SSA can be converted into GHB under the action of succinate reductase (SSR), which is reversible. GHB can also regenerate SSA under the action of GHB dehydrogenase (GHBDH) [36]. This reaction can occur in the cytoplasm, mitochondria and plastids. The specific reaction site is determined according to the type of SSR.

Similar to that of the GABA shunt, the process of GABA catabolism to form GHB is also affected by adverse environmental conditions. Stress, especially hypoxia [81], can promote the reduction of SSA to GHB and help resist stress conditions through the detoxification of SSA [82].

In this catabolic pathway, three related genes play a key regulatory role. The abovementioned *SlGABA-T2* is mainly expressed in the cytoplasm to regulate the conversion of GABA to SSA. Two *SSR* genes (*SlSSR1* and *SlSSR2*) have also been identified in tomato. *SlSSR1* is expressed in the cytoplasm, and its expression level is higher in the mature stage than in other stages. *SlSSR2* is expressed in the mitochondria and plastids, and its expression level is higher in the colour breaking stage [83]. The reductase encoded by these genes catalyses the conversion of SSA into GBH. 

**Table 1:** Key genes in the biosynthesis and catabolism of GABA in plants.

Type	Gene	Species	Description
Biosynthesis	*GAD1* [46]	Arabidopsis, Tomato, Citrus, Poplar, Tea	In Arabidopsis, it affects the GABA level of roots. In tomato, it promotes fruit growth and development but has no significant correlation with GABA. In citrus and tea, it promotes the accumulation of GABA; in poplar, there are auxin, ABA and gibberellin response elements
	*GAD2* [84]	Arabidopsis, Tomato, Citrus, Rice, Tobacco, Poplar, Tea	In Arabidopsis, it mainly affects the GABA level in the shoot but does not affect the GABA level in the root. In tomato, rice, citrus, tea and tobacco, the expression of the GAD2 gene is significantly increased, which increased the content of GABA; in poplar, there are gibberellin and ABA response elements
	*GAD3* [84]	Arabidopsis, Tomato, Tobacco, Poplar, Tea	In Arabidopsis, it has no C-terminal domain, is not regulated by Ca^2+^, and is expressed in young leaves and immature fruits. In tomato, tea, poplar and tobacco, GABA level can be increased
	*GAD4* [47]	Arabidopsis, Tomato, Poplar	In Arabidopsis, there is no effect on GABA level. In tomato, it has nothing to do with plant growth and development; in poplar, there are gibberellin response elements
	*GAD5* [85]	Arabidopsis, Poplar	It has no C-terminal domain and is not regulated by Ca^2+^ and is mainly expressed in flowers; in poplar, there are ABA response elements
	*GAD6* [86]	Poplar	There are gibberellin and ABA response elements
	*DAOs* [87]	Arabidopsis. Soybean, Peanut, Broad bean	It can oxidize Put, Spm and Spd; Cu^2+^ can activate the activity, but EDTA treatment can reduce the activity; mainly distributed in dicotyledonous plants such as legumes; Arabidopsis contains 10 CuAOs coding genes
	*PAOs* [57]	Arabidopsis, Tea, Rice, Maize, Wheat	It can oxidize Spm and Spd; FAD can be used as its coenzyme; mainly distributed in monocotyledonous plants such as cereals; Arabidopsis contains five polyamine oxidase genes (*AtPAO1-5*); tea contains seven PAO genes (*CsPAO1-7*)
Catabolism	*POP2* [63]	Arabidopsis	The production of functional GABA-T enzyme ensures the GABA gradient required to guide the growth of pollen tubes in the pistil and then regulate the development of roots and shoots
	*GABA-T1* [88]	Tomato, Poplar	In tomato, it mainly exists in mitochondria and is highly expressed, promoting the catabolism of GABA and avoiding plant dwarfing and sterility; in poplar, the expression of genes is low in leaves and increases in stems and roots in turn
	*GABA-T2* [88]	Tomato, Poplar	In tomato, it is mainly located in the cytoplasm to regulate GABA catabolism; in poplar, there is no significant difference in gene expression between leaves and stems but high expression in roots
	*GABA-T3* [80]	Tomato	Mainly expressed in plastids to promote the catabolism of GABA; ensuring the normal growth and development of plants
	*SSADH1* [17]	Arabidopsis, Tomato, Poplar	Promoting the conversion of SSA to succinate; in Arabidopsis, small size necrotic lesions of plants are avoided, and GHB production is promoted; there is no correlation with GABA content in tomato; in poplar, there are light response, gibberellin and response elements involved in anaerobic induction
	*SSADH2* [89]	Poplar	In poplar, the expression of response elements containing light response and gibberellin in leaves, stems and roots increased in turn
	*SSR1* [90]	Tomato	Promoting the conversion from SSA to GHB; it exists in the cytoplasm and has a high expression level at maturity
	*SSR2* [90]	Tomato	Promoting the conversion from SSA to GHB; it exists in mitochondria and plastids, and its expression level is high at the stage of color breaking

## 4. Transport of Exogenous GABA in Plants

GABA transporters were identified for the first time in 1999 [91]. Arabidopsis can grow efficiently on media in which GABA is the only nitrogen source, which shows that exogenous GABA can be taken up by plants [91] and verifies the existence of GABA transporters. The transport of GABA in plants includes the transport of GABA between membranes, as well as into the cell membrane to various organelles (Table 2). This process is affected by many transporters, such as aluminium activated malate transporters (ALMTs) [92], GABA transporters (GATs) [93], bidirectional amino acid transporters (BATs) [94] and cationic amino acid transporters (CATs) [95]. These transporters are located on the cell membrane or organelle membrane (Figure 1) and control the transport of GABA to the intracellular space and various organelles [93]. 

### 4.1. Transcell Membrane GABA Transporters

#### 4.1.1. ALMT1

Aluminium-activated malic acid transporters (ALMTs) are bidirectional transmembrane anion transporters [96]. Twelve *ALMT* homologous genes have been found in plants [97]. In previous studies, this protein family was shown to mainly control the transmembrane transport of malate and anions in cells. In 2018, Ramesh et al. [96] discovered that GABA can be transported across the cell membrane at a high rate under the action of ALMT1 located on the cell membrane. ALMT1 has been found in Arabidopsis, wheat, rice, rape and other plant species, and the transport of TaALMT1 to GABA has mainly been studied in wheat. *AtALMT1* and *TaALMT1* are highly homologous [98], but there has been no experiment in Arabidopsis that clearly shows that AtALMT1 transports GABA. The mechanism by which ALMT1 transports GABA is also a research hotspot at present. The function of ALMT1 in transporting GABA is closely related to the activity of anion channels. A study of GABA and malate showed that anions can activate ALMT1 [99]. Thus, there is a potential difference inside and outside the membrane, which promotes the transport of GABA [100]. Bush et al. [101,102] found that the protons generated by H^+^-ATPase passing through the plasma membrane input amino acids into the cell, while Ramesh proposed that the increased activity of ALMT can avoid the inactivation of H^+^-ATPase at the extreme hyperpolarized membrane potential, which ensures the transport of GABA and provides necessary energy [96]. Under low pH conditions, aluminium ions (Al^3+^) can promote the efflux of GABA from wheat via TaALMT1. In the case of apoplast acidification, GABA can also influx through TaALMT1. These studies suggest that pH may be the factor influencing of GABA transport [96].

Many homologous genes of the *ALMT* family have also been cloned and identified, and related protein sequences have been studied. In the process of studying related transporters, it was also found that ALMT activity decreased with increasing GABA content, indicating that GABA may negatively regulate ALMT [100]. In previous studies, Yu Long confirmed that GABA inhibits the transport of anions in wheat by changing the active structure of ALMT1 [100]. The molecular mechanism of this conformational transformation is similar to Stefano’s research on the conformational transformation of GabR combined with aspartate aminotransferase (AAT) and GABA [103]. The interaction between GABA and ALMT can be used as a plant signal to participate in the regulation of ALMT1-mediated GABA transmembrane transport.

#### 4.1.2. GAT1

GATs are a class of transcell membrane transport proteins [93,104]. The *GAT* gene belongs to the AAAP gene family [97]. Four homologous genes (*GAT1*, *GAT2*, *GAT3* and *GAT4*) have been identified in plants. GAT1 located on the cell membrane can transport GABA across the membrane and transport GABA from the apoplast to the cytoplasm. Compared with the transport of GABA by ALMT1, Al^3+^ can block the influx of GABA from the apoplast to the cytoplasm during the transport of ALMT1 [100] but has no effect on the transport of GABA by GAT1. To date, genes encoding the GAT1 protein have been identified in Arabidopsis, rice, potato and other species, and the study of *AtGAT1* transporting GABA has been carried out in Arabidopsis. Andreas et al. [93] studied AtGAT1 using *Saccharomyces cerevisiae* and *Xenopus laevis* oocytes as heterologous expression systems and found that AtGAT1 is an H^+^-driven transport protein that transports GABA through proton coupling. AtGAT1 has a very high affinity for GABA (Km10 ± 3 μM), which is the key factor in the transport of GABA.

Many studies on Arabidopsis have also verified the transport of GABA by GAT1 from other perspectives. The transient expression of AtGAT1-GFP in tobacco protoplasts showed that it localizes to the cytoplasmic membrane, which is consistent with the characteristics of GABA transport [93]. In the *AtGAT1* mutant, endogenous GABA was not affected by exogenous GABA, which compared with the WT, verifies the role of AtGAT1 in the transmembrane influx of GABA [93]. However, studies on *GAT* gene transport function in species other than Arabidopsis have not been reported, which is a valuable research direction in the future.

#### 4.1.3. AAP3 and ProT2

In the *GAT1* mutant, other quaternary transporters can partially compensate for the loss of the GAT1 transporter. Two low-affinity GABA transporters located on the cell membrane were identified by heterologous recombination in yeast, namely, amino acid permease (AAP3) and proline transporter 2 (ProT2) [105,106]. Both of these transporters are located on the cell membrane and can potentially transport GABA [107]. Genes related to these two transporters have been identified in Arabidopsis, potato, rice and other crops [108]. *AAP3* belongs to the amino acid/auxin permease (AAAP) family, and *ProT2* belongs to the amino acid transporter (ATF) superfamily [91,109]. In Arabidopsis, AtAAP3 has higher affinity for other amino acids, such as lysine, than for GABA [110]. AtProT2 has higher affinity for compatible solutions of proline and glycine betaine than GABA [107,111]. Therefore, these two low-affinity transporters can transport GABA, but the effect is not very significant.

### 4.2. Transorganelle Membrane GABA Transporter

#### 4.2.1. BAT1

BATs are bidirectional transmembrane transport proteins located on the mitochondrial membrane [94,112]. To date, seven homologous *BAT* genes have been found in Arabidopsis, potato, rice and other crop species [97,108], of which *BAT1* can transport amino acids [113]. Research on the transfer of the *BAT1* gene has only been carried out in Arabidopsis, and the gene encoding this protein in Arabidopsis (*AtBAT1*) exists as only a single copy. In the study by Bush et al., AtBAT1 had high transport activity for arginine, glutamate, lysine and other amino acids but no transport activity for GABA [94]. Michaeli found that *AtGABP* (At2g01170.1) is a splicing variant of *AtBAT1* (At2g01170) belonging to the APC gene family, mainly responsible for the transmembrane transport of GABA on the mitochondrial membrane [114]. A ^3^H-GABA experiment showed that after incubation with GABA for 10 min, the GABA divergence between the *AtGABP* mutant and WT reached 1.72 times in mitochondria, which indicated that GABP played a transport role as a mitochondrial GABA carrier. In contrast to the two low affinity GABA transporters AAP3 and ProT2 mentioned above, GABP can transport GABA but not proline with highly similar sequence structures [114]. In a study of GABP transport of GABA, it was also found that coexpression of the *GABP* gene was very highly correlated with the *SSADH* gene encoding succinate semialdehyde dehydrogenase [114], indicating that GABP may be related to GABA metabolic reactions, such as the GABA shunt and TCA cycle.

**Table 2:** GABA transport protein in plants.

Type	Transporter	Species	Description
Cell membrane	ALMT1 [100]	*Arabidopsis*, Wheat, Barley, Rice	Trans-cell membrane transport of GABA between apoplast and cytoplasm. Anions can activate its activity, and Al^3+^ can promote GABA efflux through it. GABA inhibits the transport of anions in wheat by changing the active structure of ALMT1
	GAT1 [104]	*Arabidopsis*, Rice, Potato	A high-affinity GABA transporter protein, which transports GABA from the apoplast to the cytoplasm; the *GAT1* gene belongs to the AAAP gene family
	AAP3 [91]	*Arabidopsis*, Rice, Potato	The affinity for GABA is lower than other amino acids, such as lysine; the *AAP3* gene belongs to the AAAP family
	ProT2 [111]	*Arabidopsis*, Rice, Potato	Having higher affinity for compatible solutions of proline and glycine betaine than GABA; the *ProT2* gene belongs to the ATF superfamily
Organelle membrane	CAT9 [95]	*Arabidopsis*, Tomato, Rice, Potato	Experimental verification of GABA transport function of related gene (*SlCAT9*) in tomato; the *CAT9* gene belongs to the APC gene family; transport through gradient concentration of substrate and driving force of vacuolar membrane proton pump
	GABP [114]	*Arabidopsis*	*AtGABP* (At2g01170.1) is a splicing variant of *AtBAT1* (At2g01170) belonging to the APC gene family; coexpression of *GABP* and *SSADH*

#### 4.2.2. CAT9

Cationic amino acid transporters (CATs) are located on the vacuolar membrane, and the *CAT* gene belongs to the APC gene family [95,115,116]. To date, nine homologous *CAT* genes have been found in plants, of which *CAT9* is mainly responsible for the two-way transport of GABA between the cytoplasm and vacuole. *CAT9* has been identified in tomato, potato, Arabidopsis and rice, and experimental verification of the involvement of a related gene (*SlCAT9*) in GABA transport has been carried out in tomato [95,117]. The transport of GABA by SlCAT9 is mainly realized in two ways: through the gradient concentration of the transport substrate and by the driving force of the tonoplast proton pumps on the charge exchange system. Notably, the vacuole is a special organelle, and changes in the content of amino acid components in the vacuole do not affect the osmotic pressure of the vacuole [118,119]. Therefore, all transport processes must be carried out under strict conditions. In previous research, *SlCAT9* was found to also transport Glu/Asp and may be involved in the conversion of GABA [78], thus affecting the metabolic pathway of GABA in plants. 

## 5. Function and Mechanism of GABA in the Regulation of the Abiotic Stress Response in Plants

Abiotic stress is a general term for various environmental factors that adversely affect plant growth, such as hypoxia, drought, salt, extreme temperature, heavy metal toxicity and ROS [120]. Plants accumulate a large amount of GABA under various abiotic stresses, which can carry out relevant metabolic reactions in plants according to stress type to help with stress resistance [121]. Here, on the basis of the current situation of climate and environmental change, we chose three representative types of stress, namely, hypoxic stress, salt stress and drought stress, to discuss the regulation of GABA on plant growth and development under stress and to solve the regional restrictions on plant growth since plants have limited growth areas.

### 5.1. Hypoxic Stress

#### 5.1.1. GABA Accumulation under Hypoxic Stress

Under hypoxic stress, the root system cannot absorb enough oxygen and energy from the soil or substrate, which leads to an imbalance in plant cell osmotic pressure, damages the carbon skeleton of plants and ultimately affects the growth and development of plants [122,123]. A large amount of GABA accumulates in plants under hypoxic stress [124]. For example, Yang et al. [60] showed that at the germination stage of broad bean, the GABA content of plants in the hypoxic treatment group reached 16 mg/g, 8.26 times higher than that in the control group. This phenomenon can be explained by the synthesis and degradation of GABA. During hypoxia, the release of organic acids from plant vacuoles and glycolysis to produce alanine increases the acidity of the cytoplasm and the optimal reaction pH of GAD and glutamate decarboxylation is 5.5–6.0 [125]. Therefore, the increase in the acidity of the cytoplasm can stimulate the activity of GAD and result in the synthesis of more GABA. Additionally, an acidic environment can also activate DAO to promote the degradation of polyamines to GABA [60,83]. In terms of the catabolism pathway, plant respiration is inhibited under hypoxic stress, resulting in a low ratio of NAD^+^ to NADH, which reduces the activities of SSADH and GABA-T and ultimately inhibits the catabolism of GABA, resulting in a large degree of GABA accumulation [80]. To promote GABA accumulation in response to hypoxic stress, due to different species, the synthesis and catabolism pathways may contribute concurrently, or only one of them may contribute [81].

#### 5.1.2. Function of GABA under Hypoxic Stress

In previous studies, the consumption of protons during the production of GABA under hypoxic stress was shown to regulate the intracellular pH value, and the resulting synthesized GABA enters the TCA cycle through the GABA shunt to maintain the carbon and nitrogen balance of plants and reduce the damage of hypoxic stress to plants [13]. In 2021, Wu Qi et al. [126] proposed that the increase in GABA content induced by hypoxia plays a crucial role in restoring membrane potential and preventing the interference of ROS-induced cytoplasmic K^+^ homeostasis and Ca^2+^ signal transduction, which may be achieved by the pH-dependent regulation of GABA on H^+^- ATPase or the metabolic reaction of the GABA shunt and TCA cycle [124,127]. GABA can also prevent excessive ROS accumulation and promote K^+^ efflux by controlling the *RBOH* gene and GOAK channel [128], respectively, thus improving plant resistance to hypoxic stress.

### 5.2. Salt Stress

#### 5.2.1. GABA Accumulation under Salt Stress

Currently, salt stress is considered a key factor affecting plant growth and development [129,130]. The GABA content in Arabidopsis seedlings increased 20-fold under 150 mM NaCl treatment. Many previous studies have focused on the cause of GABA accumulation under salt stress [62,131,132]. Ca^2+^ signal transduction reactions are widely recognized. That is, the intracellular Ca^2+^ concentration increases under salt stress, which promotes the combination of Ca^2+^ and Ca^2+^/CaM and then activates GAD activity to increase and synthesize GABA.

#### 5.2.2. Function of GABA under Salt Stress

GABA regulates plant growth under salt stress in multiple ways. In previous studies, a high GABA content was shown to inhibit the expression of a related salt stress response gene (*TIP2*) [133]. In recent years, the role of GABA in salt stress resistance through physiological, biochemical and molecular reactions has become a popular research topic [134,135,136]. In 2020, Ji et al. [137] found that under salt stress, the activity of GADs and GABA-Ts is activated to ensure that GABA enters the TCA cycle through the GABA shunt and increases it to resist salt damage. Su et al. [138] found that GABA can maintain membrane potential and avoid K^+^ leakage by activating H^+^-ATPase under salt stress. Cheng et al. [139] showed that GABA can alleviate salt damage during seed germination under salt-stress conditions by increasing Na^+^/K^+^ transport, promoting the accumulation of dehydration and regulating osmotic pressure. In 2020, Wu et al. [140] discovered that in the presence of exogenous GABA, the activity of antioxidant enzymes, such as superoxide dismutase (SOD), peroxidase (POD), catalase (CAT), ascorbic acid peroxidase (APX) and glutathione peroxidase (GPX), can increase to reduce the content of ROS and malondialdehyde (MDA), which is resistant to salt stress.

### 5.3. Drought Stress

#### 5.3.1. Gaba Accumulation under Drought Stress

Drought stress limits plant growth and development and leads to a reduction in plant yield [141,142]. In recent years, drought has reduced plant yield by more than 25% [143]. GABA accumulation under drought stress has multiple factors. Similar to other abiotic stresses, drought stress can also stimulate the Ca^2+^ content in plants to activate GAD activity and synthesize GABA [144]. Drought stress can induce the production of ROS in plants [145]. BC Tripathy et al. [146] found that ROS in mitochondria can activate glutamate dehydrogenase (GDH) and promote α- ketoglutarate conversion into glutamate, which can be used as the precursor of GABA, thus promoting GABA production. Drought stress can promote the degradation of polyamines into GABA according to the activity of related enzymes activated by different crops [147]. For example, in Vicia faba, PAO activity is activated, while in soybean, DAO activity is activated.

#### 5.3.2. Function of GABA under Drought Stress

GABA acts as a signalling molecule to regulate physiological and biochemical reactions in plants to increase plant tolerance to drought stress [148]. Stomatal aperture plays a key role in plant drought tolerance [77,149,150]. Stomatal guard cells contain a large amount of ALMT protein [151,152], which is the key factor in stomatal movement. In 2021, Xu et al. [153] showed that ALMT9 is the key GABA signal that regulates plant cells. Through the interaction of GABA-ALMT9, it can reduce transpiration loss and improve water use efficiency, thus resisting drought stress. Exogenous GABA could promote the accumulation of abscisic acid (ABA) in plants, which activates the ABA signal pathway and leads to stomatal closure to improve the tolerance of apple under drought stress [154]. Yong et al. [155] showed that exogenous GABA could improve the tolerance of white clover to drought by increasing the leaf water content and reducing electrolyte leakage and membrane lipid peroxidation. In addition, exogenous GABA can alleviate drought stress by maintaining membrane stability, which has been verified in perennial ryegrass and black pepper [156,157]. Furthermore, exogenous GABA induces an increase in endogenous GABA and proline; the former can maintain the normal operation of the GABA shunt and TCA cycle under drought stress, and the balance of the latter’s synthesis and catabolism is also considered to play an important role in drought stress [144].

## 6. Conclusions

GABA was found in potato tubers in 1949, and since then, it has been found to be widespread in animals and plants as a bioactive and functional compound [3]. GABA is distributed in different cell compartments, connecting multiple primary and secondary metabolic pathways, which can maintain the balance of carbon and nitrogen in plants, provide energy and regulate the pH in the cytoplasm and organelle matrix. At present, GABA is known to generally have two synthesis pathways and two catabolic pathways. However, the nonenzymatic reaction of proline discovered in recent years indicates that there are other methods of GABA synthesis in plants.

GABA transporters have been the focus of much research in recent years. Among transcell membrane transporters, ALMT1, GAT1, AAP3 and ProT2 can import GABA from the apoplast to the cytoplasm [37,53]. ALMT1 is a bidirectional transport protein that can also perform GABA efflux transport from the cytoplasm to the apoplast. Among the trans-organelle membrane transporters, GABP is a splicing variant of the bidirectional transport protein BAT1 and can transport GABA across the cytoplasm and mitochondria in both directions. CAT9 is a bidirectional transport protein located on the vacuolar membrane that can perform the bidirectional transport of GABA in the cytoplasm and vacuoles.

Compared with other stress coping methods, GABA has universal adaptability to various abiotic stresses [120]. By stimulating biosynthesis and inhibiting catabolism, GABA can accumulate to enhance the stress resistance of plants under various abiotic stresses. This process ensures that the tolerance to one stress will be improved without reducing the tolerance to another stress. On the basis of current climate conditions, many stresses often occur at the same time, such as high temperature and drought or waterlogging and flooding. Therefore, GABA, a substance capable of participating in the resistance of various abiotic stresses, can play an important role in compound stress research, crop improvement and the development of new stress resistant varieties.

This paper reviews the distribution, synthesis, catabolism, transport and stress of GABA, but there are still some unclear and unsolved problems regarding GABA. For example, some studies have noted that there is pyruvate- and glyoxylate-dependent GABA-T (GABA-TOG). However, the GABA-TOG gene has not been identified in Arabidopsis. Therefore, the existence of GABA-TOG needs further study. GABA can produce GHB under certain conditions, and some studies have shown that GHB may be related to acetyl coenzyme A and fatty acid metabolism, but what role does GHB play in plants? Ethylene is the core element for plant adaptation to hypoxia. How do the signalling pathways among GABA, ROS and ethylene interact? Further research is needed to answer these questions.
